# Dominant negative knockout of p53 abolishes ErbB2-dependent apoptosis and permits growth acceleration in human breast cancer cells

**DOI:** 10.1038/sj.bjc.6600219

**Published:** 2002-04-08

**Authors:** G C Huang, S Hobbs, M Walton, R J Epstein

**Affiliations:** Department of Medicine, King's College School of Medicine, Bessemer Rd, London, SW3, UK; CRC Centre for Cancer Therapeutics, Institute of Cancer Research, Cotswold Rd, Sutton SM2 5NG, Surrey, UK; Department of Metabolic Medicine, Imperial College School of Medicine, Du Cane Rd, London W12 0NN, UK; Division of Medical Sciences, National Cancer Centre, Hospital Drive, Singapore 169610

**Keywords:** ErbB2, p53, apoptosis

## Abstract

We previously reported that the ErbB2 oncoprotein prolongs and amplifies growth factor signalling by impairing ligand-dependent downregulation of hetero-oligomerised epidermal growth factor receptors. Here we show that treatment of A431 cells with different epidermal growth factor receptor ligands can cause growth inhibition to an extent paralleling ErbB2 tyrosine phosphorylation. To determine whether such growth inhibition signifies an interaction between the cell cycle machinery and ErbB2-dependent alterations of cell signalling kinetics, we used MCF7 breast cancer cells (which express wild-type p53) to create transient and stable ErbB2 transfectants (MCF7-B2). Compared with parental cells, MCF7-B2 cells are characterised by upregulation of p53, p21^*WAF*^ and Myc, downregulation of Bcl2, and apoptosis. In contrast, MCF7-B2 cells co-transfected with dominant negative p53 (MCF7-B2/Δp53) exhibit reduced apoptosis and enhanced growth relative to both parental MCF7-B2 and control cells. These data imply that wild-type p53 limits survival of ErbB2-overexpressing breast cancer cells, and suggest that signals of varying length and/or intensity may evoke different cell outcomes depending upon the integrity of cell cycle control genes. We submit that acquisition of cell cycle control defects may play a permissive role in ErbB2 upregulation, and that the ErbB2 overexpression phenotype may in turn select for the survival of cells with p53 mutations or other tumour suppressor gene defects.

*British Journal of Cancer* (2002) **86**, 1104–1109. DOI: 10.1038/sj/bjc/6600219 www.bjcancer.com

© 2002 Cancer Research UK

## 

The molecular pathways controlling cell growth and death are deeply intertwined, with gene products as diverse as Myc (Evan *et al*, 1992; Harrington *et al*, 1994; Barone and Courtneidge, 1995; Packham *et al*, 1996; Kauffmann-Zeh *et al*, 1997), Raf (Morrison *et al*, 1989; Pumiglia and Decker, 1997; Woods *et al*, 1997) and MAP kinase (Traverse *et al*, 1992; Ben-Levy *et al*, 1994; Marshall, 1995; Marte *et al*, 1995; Kimura *et al*, 1999) being firmly implicated in both outcomes. By the same token, well-characterised mitogens such as the epidermal growth factor receptor (EGFR) and the ErbB2 (HER2/*neu*) oncoprotein have been causally linked to cell growth inhibition and apoptosis (Gill and Lazar, 1981; Kawamoto *et al*, 1984; Filmus *et al*, 1985; Polet, 1990; Tagliabue *et al*, 1991; Armstrong *et al*, 1994; Harris *et al*, 1995; Kita *et al*, 1996). Since these molecules are often overexpressed in human breast tumours (Sainsbury *et al*, 1985; Slamon *et al*, 1989) – subtypes of which exhibit prominent apoptosis (Bodis *et al*, 1996; Liu *et al*, 1992) – a better understanding of their pathogenetic significance could be relevant to anticancer drug development.

We previously reported that growth arrest of 3T3 cells is associated with catalytic activation of ErbB2 (Epstein *et al*, 1990), and more recently demonstrated that ErbB2 lengthens and intensifies mitogenic signalling by impairing ligand-dependent EGFR downregulation (Huang *et al*, 1999). In addition, we have shown that the functionally distinct EGFR ligands, EGF and transforming growth factor-alpha (TGFα), exert differing effects on EGFR downregulation and hence on the duration of ErbB2 co-activation: high concentrations of EGF initially cause prolonged EGFR activation associated with ErbB2 heterodimerisation, followed by eventual EGFR downregulation and signal cessation; whereas TGFα fails to downregulate EGFR, leading to sustained signalling (Gulliford *et al*, 1997; Ouyang *et al*, 1999a). The possibility is thus raised that ErbB2 could mimic the tumorigenic effects of TGFα in cancer cells by its similar ability to prolong EGFR signalling.

The above-mentioned differential induction of growth stimulation or inhibition by EGFR (Filmus *et al*, 1985; Polet, 1990; Armstrong *et al*, 1994; Gulli *et al*, 1996) and ErbB2 (Tagliabue *et al*, 1991; Harris *et al*, 1995; Kita *et al*, 1996) strongly suggests an interaction between downstream signal duration (e.g. of MAP kinase) and cell cycle control proteins (Traverse *et al*, 1992; Marshall, 1995). To address the possibility that ErbB2-dependent changes in signal duration may contribute to such differences in cell fate, it is necessary to create cell systems in which the effects of ErbB2 expression can be correlated with the function or dysfunction of a given cell cycle regulatory molecule. Here we show that the effects of ErbB2 on cell signalling kinetics are selectively associated with induction of apoptosis in oestrogen-responsive MCF7 human breast cancer cells – which, like most hormone-sensitive cancers (Caleffi *et al*, 1994; Elledge *et al*, 1995; Berns *et al*, 2000), express wild-type p53 (Casey *et al*, 1991; Balcer-Kubiczek *et al*, 1995; Furuwatari *et al*, 1998) but normally do not overexpress ErbB2 (Wright *et al*, 1997; Ferrero-Pous *et al*, 2000; Pinto *et al*, 2001). Dominant negative knockout of p53 converts growth inhibition to growth enhancement in these ErbB2-transfected cells, suggesting that a p53 mutational pathway could favour selection for ErbB2 gene amplification during tumour progression.

## MATERIALS AND METHODS

### Cell lines, reagents, antibodies, and immunoblotting

MCF7 and A431 cells were obtained from the American Type Culture Collection (Rockville, MD, USA). Synthetic human EGF and TGFα were purchased from Sigma. Activation-state-specific EGFR antibodies, and antibodies to p53, Myc, Bcl2 and p21^*WAF*^, were purchased from Cambridge BioScience (Cambridge, UK). Polyclonal antibodies to Tyr^1248^- and Tyr^1222^-phosphorylated ErbB2 were developed and validated for receptor-specificity as described previously (Epstein *et al*, 1992; Ouyang *et al*, 1998). For immunoblotting studies, cells were lysed as previously described (Gulliford *et al*, 1997): protein lysates were immediately boiled for 5 min in sample buffer (6.7% sodium dodecyl sulfate, 30% glycerol, 62.5 mM Tris base pH 6.8, 0.01% bromophenol blue) then loaded onto a 7.5% SDS-polyacrylamide gel. Samples were electrophoresed and transblotted onto nitrocellulose as described (Towbin *et al*, 1979).

### Growth curves and apoptosis assays

Cell growth was measured using a multiwell colorimetric assay based on sulphorhodamine B (SRB) spectrophotometric detection. Confirmation and quantification of morphologic apoptosis was performed using a Tdt-mediated dUTP nick-end labelling (TUNEL) kit to directly detect DNA fragmentation *in situ*. Briefly, cells were plated and grown on glass slides, treated with ligand for the required period, then fixed in 4% paraformaldehyde for 30 min at room temperature. The slides were washed with PBS three times, after which the cells were permeabilised with 0.1% Triton-X-100 in 0.1% sodium citrate for 10 min. After washing, the cells were covered in 50 μl of equilibration solution for 10 min, then covered with 50 μl of labelling solution (Biovation) and incubated at 37°C for 1 h while light-protected. The slides were then washed, covered in 10 μl counterstain for 10 min, and analysed using fluorescence microscopy.

### Cell transfection

For calcium phosphate transfection, cells were seeded in 90 mm diameter cell culture dishes at 5×10^5^ cells ml^−1^ 24 h before the transfection. One plate was required for each transfection experiment; the monolayer normally grew to 80% confluence by the following day, and the medium was changed 3 h before the transfection. Two sterile microfuge tubes were labelled for each transfection experiment: to one tube was added 500 μl of 2×BBS (pH 6.95) and to the other tube was added 125 μl of 1 M CaCl_2_, 10–20 μg of recombinant plasmid DNA which contained the relevant cDNA; distilled H_2_O was added to give a final volume of 500 μl. This was added to equal the volume of 2×concentrated BBS using a sterile Pasteur pipette. At the same time, filtered air was passed through the 2×BBS buffer (pH 6.95) with a second Pasteur pipette, and the DNA mixture was then incubated at room temperature for 20 min to allow precipitation. The DNA/CaPO_4_ precipitate was mixed by inverting the tube, and was added directly to a 10 ml cultured cell dish dropwise with gentle shaking, and the cell culture incubated at 37°C with 3% CO_2_ overnight followed by washing with PBS and re-culturing in fresh medium at 37°C with 5% CO_2_.

### Constructs and selection procedures

The well-characterised temperature-sensitive dominant negative p53 construct (Kuerbitz *et al*, 1992; Slichenmeyer *et al*, 1993; Zhang *et al*, 1994; Vasey *et al*, 1996) was kindly provided by Dr B Vogelstein (Baker *et al*, 1990). For selection, transfected cells were plated at 5×10^4^ cells/9 cm tissue culture dish with relevant reagents: dominant negative p53 was selected with neomycin. The wild-type ErbB2 construct, which is under the control of the Moloney murine leukaemia virus LTR and contains the *Ecogpt* selectable marker from *E. coli* (Di Fiore *et al*, 1987), was selected with HAT (hypoxanthine, aminopterin and thymidine) as described by Mulligan and Berg (1981). For double transfection a pool of six p53 dominant negative clones (Δp53) or p53 empty vector clones were transfected with either ErbB2 or ErbB2 empty vector, and selected with HAT medium for at least 6 weeks. Resistant colonies were cloned and a pool of six clones was cultured with HAT medium to amplify the cell number. For analysis, the cells were cultured in normal medium for at least 2 weeks before the experiments were performed. For morphologic analysis, cells were grown in plastic 8-chamber containers (LabTek; Gibco) and the monolayers photographed using a Zeiss microscope. Growth experiments were carried out in 96-well plates using quantification of Hoechst dye immunofluorescence in six matched samples following 3 days growth to assess cumulative DNA content.

## RESULTS

**Figure 1:**
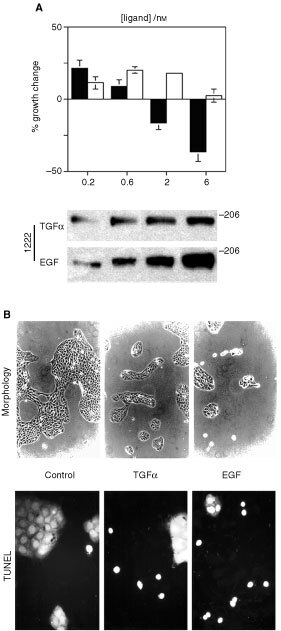
Relative effects of EGF and TGFα on cell growth, growth inhibition, and ErbB2 tyrosine phosphorylation of A431 cells. (**A**) Effects of differential ligand treatment on cell growth, relative to growth of untreated controls (upper figure) and to ligand-dependent ErbB2 tyrosine phosphorylation (lower figure). In the upper figure, cells were plated at 1.5×10^4 ^ml^−1^ seeding density and stimulated for 6 days with the respective ligand (EGF, solid columns; TGFα, open columns) prior to counting using a sulphorhodamine-based assay as described. Nanomolar ligand concentrations are represented on the abscissa. The results are expressed as a percentage change relative to control cell growth; error bars are based on six measurements. The lower figure shows the corresponding short-term effects of EGF and TGFα on ErbB2 Y^1222^ phosphorylation: cells were treated for 5 min with EGF or TGFα at the indicated nanomolar concentration prior to lysis, electrophoresis and immunoblotting using aPY^1222^. The bands were visualised using ECL. (**B**) Visualisation of cell death by light microscopy and TUNEL assay (see Materials and Methods) associated with ligand treatment. Twenty-four hours following attachment, cells were treated with the respective ligands (2 nM) in serum-free medium. Typical low-power views of triplicate plates are shown after 48 h treatment using light microscopy (above) and fluorescence microscopy (below).

Consistent with earlier reports (Gill and Lazar, 1981; Polet, 1990), ligand stimulation experiments confirm EGF-dependent growth inhibition of sparsely-plated A431 cells (Figure 1A

, upper panel). The extent of growth inhibition correlates with the intensity of equimolar ligand-dependent ErbB2 tyrosine phosphorylation as detected by site-specific phosphoantibodies (Ouyang *et al*, 1998) which confirm greater ErbB2 tyrosine phosphorylation following EGF stimulation (Figure 1A, lower panel). As reported previously, this initial difference in ligand-dependent signal intensity is maintained and further exaggerated over the subsequent 12 h (Ouyang *et al*, 1999a). Correlation of light microscopy with TUNEL assay indicates that the growth-inhibitory effects of EGF in this context are associated with increased apoptosis (Figure 1B).

**Figure 2:**
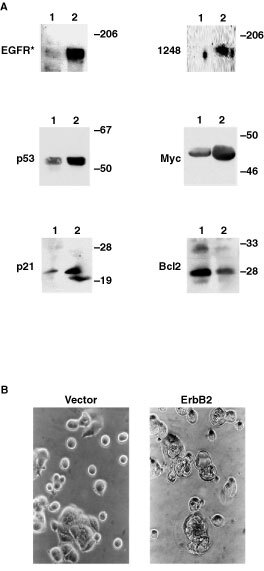
Effects of transient ErbB2 transfection on parental MCF7 cells. (**A**) Effects of ErbB2 transfection on protein expression. Control (lane 1; vector-only) and ErbB2-transfected cells (lane 2) were lysed and assessed by immunoblotting. EGFR*, kinase-active epidermal growth factor receptor; 1248, ErbB2 tyrosine-phosphorylated at position 1248, detected by the activation-specific aPY^1248^ antibody (Ouyang *et al*, 1998). (**B**) Effects of ErbB2 expression on morphology and apoptosis of MCF7 human breast cancer cells assessed using light microscopy. ErbB2 transfectants (at right) were generated using a standard calcium phosphate transfection procedure followed by neomycin selection. Mock transfectants containing empty plasmids are shown at left.

The foregoing data do not distinguish whether the observed growth inhibition is induced by ligand-dependent ErbB2 co-activation *per se* or, alternatively, by the downstream consequences of growth factor signal prolongation induced by ligand-dependent ErbB2 heterodimerisation. However, since our previous work documented a marked prolongation of EGFR signalling by ErbB2 expression (Huang *et al*, 1999), we elected to test the latter hypothesis by creating ErbB2 transfectants in cell lines differing solely in terms of cell cycle control functionality. To this end, MCF7 human breast cancer cells known to express both copies of the wild-type p53 gene (Casey *et al*, 1991; Balcer-Kubiczek *et al*, 1995; Furuwatari *et al*, 1998) were transiently transfected with ErbB2. As shown in Figure 2A

, ErbB2 expression in these cells induces increased immunoreactivity of both activated ErbB2 and EGFR, consistent with previous studies (Huang *et al*, 1998, 1999), while also inducing increased expression of p53, p21^*WAF*^ and Myc. Of note, ErbB2 expression is associated with reduced *Bcl*2 expression – an effect reported previously following primary overexpression of p53 (Haldar *et al*, 1994). These effects on protein expression are accompanied by morphologic changes (membrane blebbing, chromatin condensation) typical of apoptosis in ErbB2-transfected, but not vector control, cells (Figure 2B). These ErbB2-dependent changes in protein expression and morphology directly implicate ErbB2 in the activation of an apoptotic pathway.

**Figure 3:**
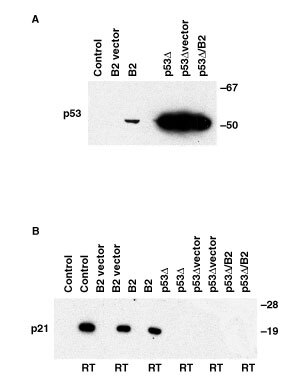
Effects of stable ErbB2 overexpression and/or p53 knockout on MCF7 cell protein function as measured by expression of p53 and p21^*WAF*^ in ErbB2 and/or mutant p53 transfectants and controls. (**A**) p53 immunoblot. Control, parental MCF7 cells; B2 vector, MCF7 transfected with vector alone; B2, MCF7 transfected with vector containing ErbB2 cDNA; p53Δ, MCF7 transfected with dominant negative mutant p53; p53Δ vector, MCF7 transfected with dominant negative p53 and also with empty vector used for ErbB2 studies; p53Δ/B2, MCF7 cells transfected with dominant negative mutant p53 and with ErbB2. *B*, p21^*WAF*^ immunoblot. As indicated by the legend below, cells from even-numbered lanes received radiotherapy (RT) with 0.1 Gy X-irradiation prior to lysis. Odd-numbered lanes represent the sample order described for the upper panel.

To clarify whether the apoptosis-triggering effect of ErbB2 might be at least partly related to its effects on signalling kinetics (i.e. as opposed to an exclusive cell-killing effect of ErbB2 kinase activity), stable MCF7 cell transfectants were created using either the wild-type ErbB2 gene, the dominant-negative p53 mutant gene, or both. As in the ErbB2 transient transfectants, stable overexpression of ErbB2 selectively induces endogenous (wild-type) p53 protein overexpression (Figure 3A

, upper panel, left three lanes); as expected, dominant-negative mutant p53 (Δp53) cells grossly overexpress immunoreactive p53 (Figure 3A, upper panel, right three lanes). Irradiated control and ErbB2-transfected MCF7 cells exhibit a normal increase in p21^*WAF*^ expression following X-irradiation (Figure 3B, lower panel, left 6 lanes). In contrast, MCF7-Δp53 cells sustain no immunodetectable rise in p21^*WAF*^ levels (Figure 3B, lower panel, right 6 lanes), thus validating the functionality of the dominant-negative p53 construct used in these experiments. Of note, p21^*WAF*^ was not detectably overexpressed in stable ErbB2-overexpressing cells (Figure 3B) unlike in transient transfectants (Figure 2A), raising the possibility that prolonged ErbB2 overexpression induces clonal selection.

**Figure 4:**
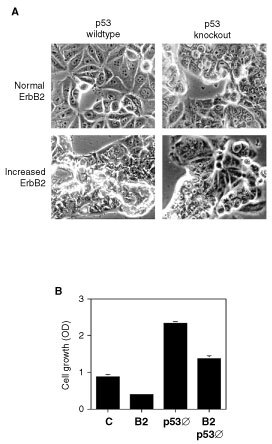
Effects of ErbB2 overexpression and/or p53 knockout on MCF7 cell morphology and growth. (**A**) Morphology of MCF7 cell variants characterised by light microscopy. Top left, parental MCF7 cells; top right, MCF7 transfected with mutant p53; lower left, MCF7 transfected with wild-type ErbB2; lower right, MCF7 co-transfected with both mutant p53 and wild-type ErbB2. (**B**) Cell growth of MCF7 variants following 3 days growth. Error bars represent standard errors of the mean based on six identical samples for each cell line. Abbreviations are as above.

The four transfectant cell lines of interest – parental MCF7, MCF7-B2, MCF7-Δp53, and MCF7-B2/Δp53 – were then compared with respect to morphology and growth. Unlike parental MCF7 cells which adopt a spread-out cell appearance suggesting density-dependent growth inhibition (Figure 4A

, upper left), all of the other transfectants exhibit a crowded morphology. MCF7-B2 cells also exhibit striking apoptosis (Figure 4A, lower left), however, a feature which is absent from both the MCF7-Δp53 and MCF7-B2/Δp53 cells (Figure 4A, right upper and lower panels, respectively). Cell growth as measured by Coulter counting was increased in MCF7-Δp53 cells and reduced in MCF7-B2 cells relative to parental cell growth: MCF7-B2/Δp53 cells exhibit more rapid growth than parental cells, though slower than MCF7-Δp53 cells (Figure 4B). Given the foregoing results, these data indicate that the observed ErbB2-dependent effects on cell fate vary with the functional status of p53, suggesting in turn that p53 may act as a sensor for ErbB2-induced changes in cell signalling kinetics.

## DISCUSSION

We previously showed that ErbB2 expression causes constitutive EGF signalling by retarding downregulation of hetero-oligomerised EGFR (Huang *et al*, 1999). This effect most likely relates to the absence of motifs in the ErbB2 C-terminal tail for receptor internalisation and degradation (Sorkin *et al*, 1993; Baulida *et al*, 1996). Since human tumours exclusively overexpress the wild-type ErbB2 rather than the transforming point mutant (Lemoine *et al*, 1990), a reasonable hypothesis is that tumour cells acquire a growth advantage from wild-type ErbB2 overexpression, but that this phenotype does not represent the primary transforming event – implying the co-existence, that is, of at least one other molecular defect within the tumour cells. This hypothesis is consistent with numerous reports linking tumour cell ErbB2 overexpression and p53 dysfunction (Horak *et al*, 1991; Mehta *et al*, 1995; Li *et al*, 1997) and identifying poor-prognosis clinical subgroups based on concurrence of these phenotypes (Tsuda *et al*, 1998). Moreover, our recent documentation of differential survival outcomes in ErbB2-overexpressing breast cancers associated with different phosphorylation patterns (Ouyang *et al*, 1999b, 2001) supports the notion of multiple signalling pathways governing tumour growth phenotypes.

Given that the p53 checkpoint prevents cell-cycle progression when activated (Casey *et al*, 1991; Yin *et al*, 1992; Wyllie *et al*, 1995) and that the duration of growth factor signalling influences whether cells proliferate or arrest (Traverse *et al*, 1992; Marshall, 1995), the present study suggests a model of cell signal sensing which is differentially perturbed by ErbB2 depending upon the functional p53 status. Other studies have concluded that the main *in vitro* and *in vivo* consequences of p53 mutation on cell growth relate to enhanced proliferation rather than to reduced apoptosis (Nikiforov *et al*, 1996; Tyner *et al*, 1999). Our data suggest a more complex interpretation of p53 function as a co-variable within the cell growth machinery; this is consistent with the surprising finding in human tumours that p53 mutation is often associated with increased, rather than decreased, apoptotic indices (van Slooten *et al*, 1999). In the context of tumour progression, it is important to note that apoptosis could represent a mechanism of clonal selection for more aggressive cell lineages, rather than simply indicating a benign tumour-suppressive function.

Reductions in mitogenic signal intensity may normally cause cells to arrest and/or differentiate, whereas signal prolongation may trigger differentiation or death (Traverse *et al*, 1992; Dolmetsch *et al*, 1997). According to this paradigm, apoptosis may be inducible by forced cell cycle progression in the presence of activated checkpoints (Polet, 1990). Abrogation of p53 function by mutation could thus prevent cells from sensing an abnormally prolonged signal, leading to loss of growth arrest, reduced apoptosis and differentiation, and consequent outgrowth of less differentiated cells. In contrast, ErbB2-dependent impairment of EGFR downregulation both prolongs and intensifies growth factor signalling (Huang *et al*, 1999), an outcome associated with the increased apoptosis reported here. Such an effect of ErbB2 might be expected to be short-lived, given that selection for apoptotic resistance should be rapid (Balcer-Kubiczek *et al*, 1995). Acquisition of a p53 defect in this context would cause mutant cells to ‘perceive’ mitogenic signals as short despite ErbB2-dependent signal prolongation – leading to apoptotic resistance, dedifferentiation and clonal outgrowth.

Human tumours could thus evolve from an interplay between progressive ErbB2 overexpression and acquisition of cell-cycle control defects including, though not necessarily limited to, p53 mutations. We therefore submit that human tissues with cell-cycle control defects (De Cremoux *et al*, 1999; Prevo *et al*, 1999) may gain a growth advantage by prolonging and intensifying ambient growth factor signals via ErbB2 upregulation, and that tumour cells overexpressing ErbB2 may in turn clonally select for cell-cycle checkpoint loss (Li *et al*, 1997).
